# Retinal abnormalities in universal eye screening of healthy, full-term newborn infants in Jakarta. The incidence and its risk factors: a pilot study

**DOI:** 10.1186/s40942-021-00337-1

**Published:** 2021-11-03

**Authors:** Rita S. Sitorus, Indra Maharddhika Pambudy, Rinawati Rohsiswatmo, Julie Dewi Barliana, Dian Estu Yulia, Indah Suci Widyahening

**Affiliations:** 1 Department of Ophthalmology, Faculty of Medicine Universitas Indonesia, Cipto Mangunkusumo National Referral Hospital, Jalan Kimia No. 8-10, Jakarta, 10320 Indonesia; 2 Department of Child Health, Faculty of Medicine Universitas Indonesia, Cipto Mangunkusumo National Referral Hospital, Jakarta, Indonesia; 3 Department of Community Medicine, Faculty of Medicine Universitas Indonesia, Jakarta, Indonesia

**Keywords:** Universal eye screening, Hemorrhage, Delivery, Healthy newborn

## Abstract

**Aim:**

To screen for ocular abnormalities in healthy full-term newborn infants using wide-field digital imaging and to analyze factors associated with the findings.

**Methods:**

A total of 1208 full-term newborn infants at a tertiary eye hospital (Cipto Mangunkusumo National Referral Hospital) and a district hospital in Jakarta (Koja Hospital) were enrolled to the study. All eligible newborns underwent fundus examination within 48 h after birth using the RetCam shuttle (Natus Medical Incorporated, USA). Retinal findings were documented and analyzed according to obstetric and neonatal risk factors.

**Results:**

Of the 1208 newborn infants enrolled, ocular abnormalities were found in 150 infants (12.4%). Retinal hemorrhage (RH) was the most common finding (88%) in which 2.67% involved the macula, followed by chorioretinitis (4.67%). Univariate analysis showed caesarean section (C-section) (OR 0.27, 95% CI 0.18–0.41, p < 0.001) was a protective factor against RH, while prolonged labor increased the risk of developing RH (OR 1.84, 95% CI 1.24–2.72, p = 0.002). Further multivariate analysis showed similar protective association between C-section and risk of RH (OR 0.29, 95% CI 0.19–0.44, p < 0.001), while other risk factors were not.

**Conclusions:**

Our study showed that universal eye screening in healthy neonates is beneficial in the early diagnosis, monitoring and treatment of ocular abnormalities such as retinal hemorrhage, chorioretinitis and retinoblastoma. Retinal hemorrhage is the most common ocular abnormality and is associated with the delivery method and the duration of labor. Universal eye screening is visual-saving and life-saving for neonates with chorioretinitis, retinoblastoma as well as other abnormalities and should be mandatory in newborn screening.

## Background

Many ocular abnormalities that occur at birth could lead to permanent visual loss. Therefore, it is necessary to identify any eye problems as early as possible since it could significantly impact child development [].

Newborn eye screening is not yet standard protocol in many countries although several methods have been used to examine for eye diseases at birth. The American Academy of Pediatrics recommends Red Reflex Testing (RRT) for every newborn. The RRT is a photo-screening test using digital camera-like equipment, which has been used as a screening method for young children aged one to five [1]. Universal eye screening in healthy newborns using a wide-field digital camera has been studied in different countries to detect early ocular abnormality [2, 3].

The reported incidence of newborn retinal hemorrhage varies widely from 2.4 to 34% [2–7]. Emerson et al. [6] reported that retinal hemorrhage (RH) occurs more frequently in newborns delivered by vacuum extraction (75%), followed by spontaneous vaginal delivery (33%), and C-Section (6.7%) [7].

Currently, the exact causes and risk factors of newborn RH remain unclear. This study aims to screen for ocular abnormalities in healthy full-term newborn infants using wide field digital imaging and to investigate the risk factors contributing to RH. This is the first universal eye screening study of healthy newborn infants reported in Indonesia.

## Methods

A cross-sectional study was conducted in two different hospitals, Cipto Mangunkusumo National Hospital (CM Hospital), the national referral hospital of Indonesia, and Koja Hospital, a district hospital in Jakarta, from January to June 2015. Inclusion criteria were healthy, full term, newborn infants aged < 48 h and the exclusion criteria were those with red eyes prior to the examination, or whose parents declined to participate in this study. Written informed consent was given by each parent prior to the examination of their infant. This study is in accordance with The Declaration of Helsinki and received ethical clearance from the Ethics Committee of Faculty of Medicine, Universitas Indonesia, CM Hospital.

The eye screening was performed within the first 48 h after birth in a room with trained technicians assisted by a neonatal nurse. The anterior segment was evaluated using non-contact wide-angle portrait camera lens. Thereafter, the pupil was dilated by tropicamide 0.5% and phenylephrine 2.5%. Topical anesthetic eye drops were instilled shortly before the examination. After which, wide-angle digital fundus camera examination was performed using the Retcam (RetCam Shuttle, Natus Medical Incorporated, USA) first in one eye and then the other. A minimum of five photos were taken for each eye: the posterior pole, superior, inferior, nasal, and temporal retinal fields. Topical antibiotic was given in each eye when the procedure is completed. Heart rate, oxygen saturation and general condition of patients were observed for 24 h after completion of procedure to monitor for adverse effects of topical mydiatrics.

Referral to the eye clinic of CM Hospital was recommended for patients whose eye exam demonstrated an abnormal finding.

In bilateral cases, the eye with worse degree of RH was included. Retinal hemorrhage was graded according to Egge’s classification [8] as seen in Table 1.

**Table 1:** Egge’s Classification of Retinal Hemorrhage

Grades	Definition
Grade I	Small, mainly less than a quarter of the disc diameter and relatively few hemorrhages in one or both eyes
Grade II	Medium to large hemorrhages which were not exceeding the optic disc diameter in size, or a combination of few such hemorrhages and many smaller ones in one or both eyes
Grade III	Larger hemorrhages, the diameter of which were larger than that of the optic disc, in one or both eyes and sometimes also combined with smaller and/or larger apparently pre-retinal hemorrhages

All imaging procedures were done by two general practitioners trained in using the RetCam Shuttle device who were aware of the safety precautions in handling neonates. The captured images were then analyzed by three pediatric ophthalmologist consultants. In case of discrepancies among the consultants, discussion regarding the image(s) was made to establish a final diagnosis. All findings were documented in an excel sheet along with patient details.

Data analysis was performed using the Statistical Package for Social Sciences (SPSS) for Mac version 10.5. The association between RH to gender, delivery method, and prolonged labor were assessed using univariate analysis. To prevent a type 2 error, only variables with a univariate p-value of 0.20 or less were selected for further multivariate analysis. Factors with a p-value less than 0.05 were considered statistically significant.

## Results

A total of 1208 full-term newborns were examined in two public hospitals in Jakarta, and the results were as follows.

Table 2 shows the demographic data of patients. Most of the newborn infants were female (55.28% and 50.26% in CM and Koja Hospital respectively) and the mean birth weight in CM Hospital was 2663 g and 2978 g in Koja Hospital. The mean duration of latent and active phase during first stage labor were 518 ± 254 min and 238 ± 124 min, respectively in CM Hospital. Meanwhile in Koja Hospital, the mean of first labor latent phase was 574 ± 520 min and first labor active phase was 280 ± 250 min. Prolonged labor occurred in 11.2% and 31.6% patients in CM and Koja Hospital respectively. More than half of the mothers in CM Hospital (64, 68%) and nearly half (45%) of mothers in Koja Hospital underwent C-section. There were 14 patients in CM Hospital (3.1%) and 2 patients in Koja Hospital (0.26%) who underwent combined delivery method (C-section with forceps/vacuum instrumentation). The mean 1- and 5-min APGAR scores were 8/9 and 7/8 in CM and Koja Hospital, respectively (Table 3).

**Table 2:** Demographic data

	CM hospital (N^a^ = 436)	Koja hospital (N^a^ = 772)
Newborn infant characteristics		
Gender, n (%)		
Female	241 (55.3)	388 (50.3)
Male	195 (44.7)	384 (49.7)
Birth weight (grams)	2663 (SD ± 659)	2978 (SD ± 526)
Prolonged labor	49 (11.2)	244 (31.6)
Methods of delivery		
Spontaneous vaginal delivery, n (%)	128 (29.4)	400 (51.8)
C-section, n (%)	282 (64.7)	348 (45.1)
C-section + forceps, n (%)	13 (3.0)	2 (0.3)
C-section + vacuum, n (%)	1 (0.2)	0 (0)
Forceps, n (%)	6 (1.4)	1 (0.1)
Vacuum, n (%)	5 (1.1)	21 (2.7)
Forceps + vacuum, n (%)	1 (0.2)	0 (0)
APGAR score 1 min (mean)	8	7
APGAR score 5 min (mean)	9	8

^a^Total number of patients

**Table 3:** Results of newborn eye examination

Ocular abnormalities	CM Hospital		Koja Hospital	
	N^a^ = 436	%	N^a^ = 772	%
Retinal hemorrhage	30	6.9	102	13.1
Macular hemorrhage	0	0	4	0.5
Optic nerve head hemorrhage	0	0	1	0.1
Localized blood in the vitreous cavity/peripapillary hemorrhage	0	0	1	0.1
Chorioretinitis/exudation requiring systemic examination	1	0.2	6	0.8
Macular dystrophy/maculopathy	0	0	1	0.1
Intraocular tumor (retinoblastoma)	0	0	1	0.1
Persistent pupillary membrane or persistent tunica vasculosa lentis	0	0	1	0.1
Optic neuropathy dd/coloboma optic	0	0	1	0.1
Iris nodule	1	0.2	0	0

^a^Total number of patients

Examination of the anterior segment did not show any abnormalities. The infants showed stable heart rate and oxygen saturation with good general condition within 24 h after procedure without systemic complications from topical mydriatics.

**Fig. 1:**
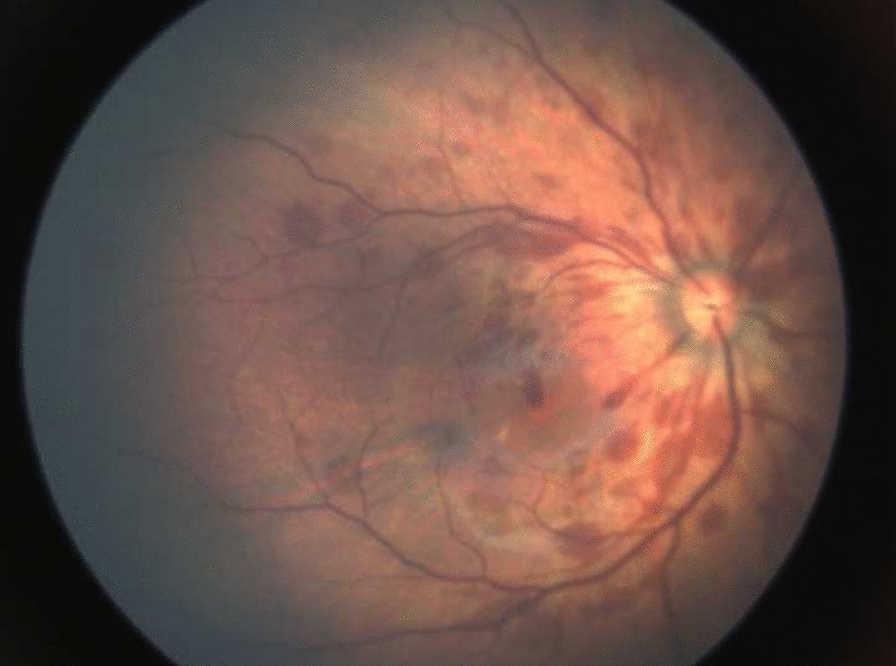
Retinal hemorrhage grade III with flame shape pattern involving macular area

**Fig. 2:**
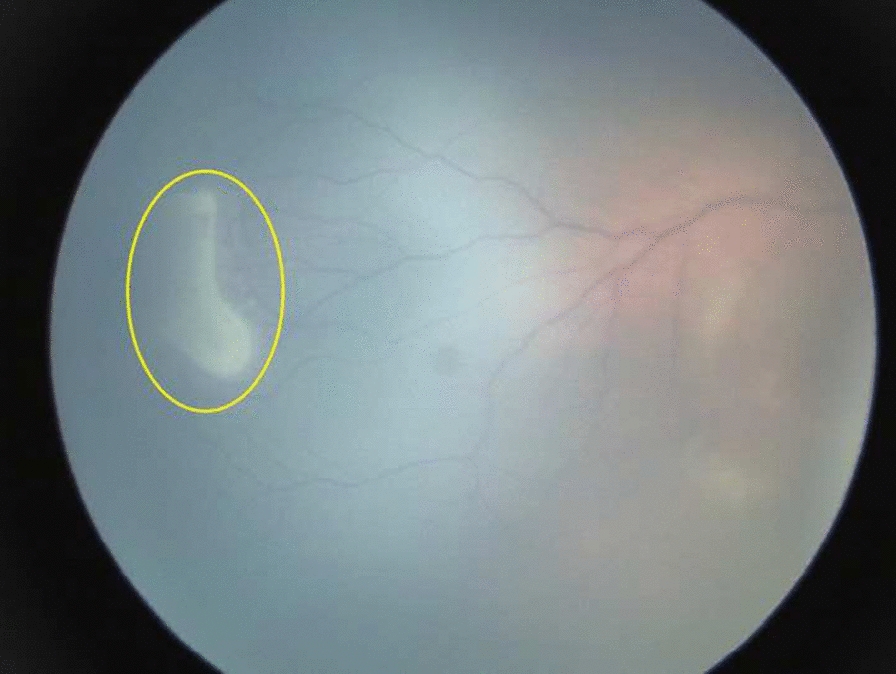
White exudative lesion involving temporal peripheral quadrant of the retina, as indicated by the yellow circle

**Fig. 3:**
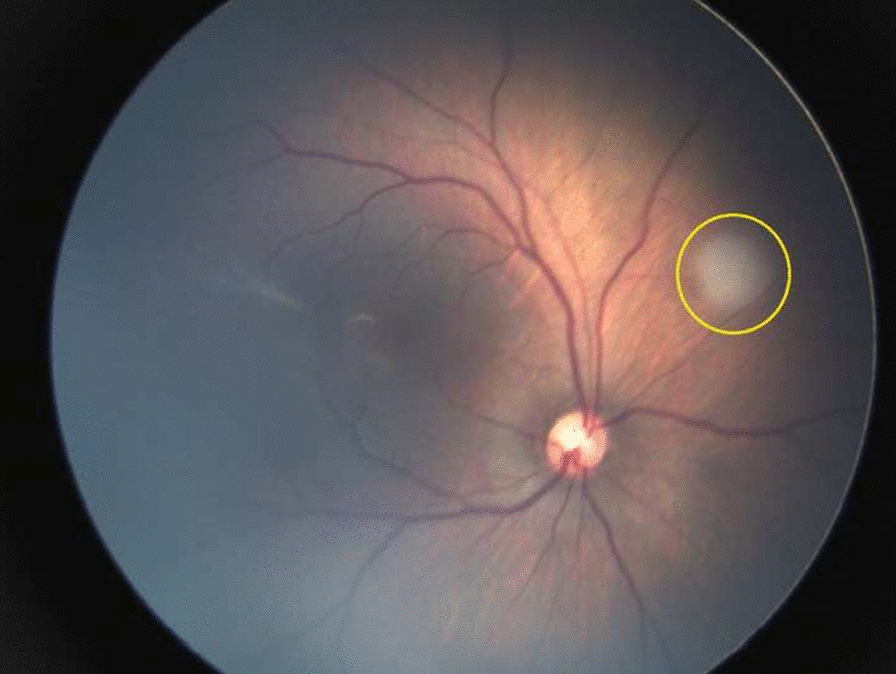
White mass at supero-nasal quadrant of the retina, suspected of intraocular tumor (retinoblastoma), as indicated by the yellow circle

**Fig. 4:**
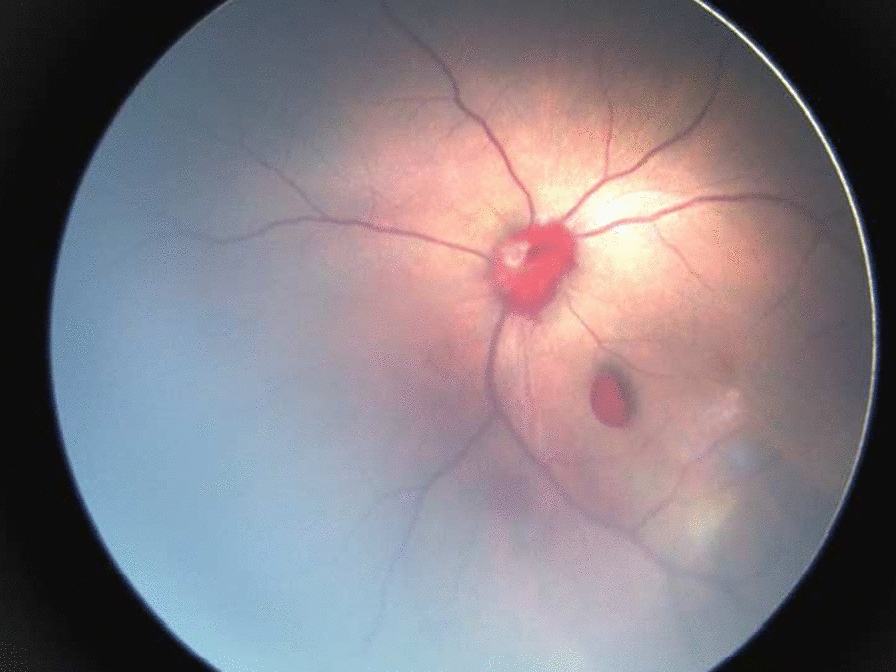
Localized blood in the vitreous cavity, peripapillary hemorrhage

Among the infants examined, 32 (7.3%) infants in CM Hospital and 118 (34.9%) infants in Koja Hospital had ocular abnormalities as summarized in Table 3. Retinal hemorrhage (Fig. 1) was the most common ocular abnormality observed, found in 6.88% and 13.11% newborns in CM Hospital and Koja Hospital, respectively. Chorioretinitis (Fig. 2) was the second most common ocular abnormality found (0.57%), followed by macular hemorrhage (0.33%). Other ocular abnormalities such as retinal exudate, maculopathy, intraocular tumor (suspected retinoblastoma shown in Fig. 3), optic nerve head abnormality, iris nodule, persistent pupillary membrane, and localized blood in the vitreous cavity/peripapillary hemorrhage (Fig. 4) were found in 1 participant (0.08%) (Table 2).

Among cases of retinal hemorrhages found, 64 infants (48.5%) had grade III RH, 59 infants (44.7%) had grade II RH while nine infants (6.8%) had grade I RH (see Fig. 1).

Table 4 demonstrates the risk factor analysis of retinal hemorrhage in neonates. Univariate analysis showed that C-section delivery method reduced the risk of RH (OR 0.27, 95% CI 0.18–0.41, p < 0.001) while prolonged labor increased the risk of RH (OR 1.84, 95% CI 1.24–2.72, p = 0.002). Both gender and instrument-assisted delivery showed no significant association towards RH (p > 0.05). Furthermore, C-section was found to be a protective factor against RH in multivariate analysis (OR 0.29, 95% CI 0.19–0.44, p < 0.001) while other risk factors were not. APGAR score was not significantly associated with the incidence of retinal hemorrhages in either univariate or multivariate analysis.

**Table 4:** Risk factor analysis of retinal hemorrhage

Variables	Univariate analysis [OR (CI 95%)]	P value	Multivariate analysis [OR (CI 95%)]	P value
Gender (Female)	0.74 (0.52–1.07)	0.11	1.33 (0.92–1.92)	0.13
Methods of delivery				
C-section^a^	**0.27 (0.18–0.41)**	< 0.001*	**0.29 (0.19–0.44)**	< 0.001*
Delivery method with instrument (vacuum, forceps, or both)^a^	0.78 (0.34–1.79)	0.561	0.73 (0.32–1.69)	0.47
Prolonged labor^b^	**1.84 (1.24–2.72)**	**0.002**^#^	0.81 (0.54–1.23)	0.33
APGAR Score	0.796 (0.88–1.99)	0.183	1.870 (1.19–2.93)	1.87

^a^Compared to spontaneous vaginal delivery

^b^Prolonged labor is defined as prolonged duration of delivery at any stage (stage 1, stage 2, or combined)

*Odds ratio of less than 1 and p<0.05 indicate that C-section is a significant protective factor against RH ^#^Odds ratio greater than 1 and p < 0.05 indicate that prolonged labor is a significant risk factor for RH

## Discussions

In most developing countries including Indonesia, universal eye screening of the newborn is not a common practice. However, techniques and technologies in retinal examination have improved and become more readily available during the last few decades. This would facilitate earlier diagnosis of ocular abnormalities among newborns in Indonesia.

During our study, we found that there was a difference in delivery pattern between our two hospitals. In CM Hospital, the rate for caesarean section was 64.7% compared to that of Koja hospital (45.1%). This difference is because CM Hospital is the top referral hospital in Jakarta that predominantly managed high-risk pregnancies in which caesarean section was often indicated. On the other hand, Koja Hospital is a district hospital which handled simpler, less complicated cases where spontaneous delivery can be attempted, explaining its lower rate of caesarean section compared to CM hospital.

In our study, we screened a total of 1208 healthy newborns and found that RH (Fig. 1) was the most common ocular abnormalities (10.93%), followed by chorioretinitis (0.58%) and macular hemorrhage (0.33%). Other ocular abnormalities such as macular dystrophy, intraocular tumor, optic nerve head abnormality, iris nodule, and persistent pupillary membrane were the least common abnormalities (0.08% each).

We found that seven out of 150 patients with ocular abnormalities, were suspected to have chorioretinitis. Exudative retinal lesions found in this study was most likely due to intrauterine infection because the lesion is in the perivascular area (as seen in Fig. 2). Serological test would be required to confirm the diagnosis, however, this data was unavailable as it was not the standard diagnostic procedure during antenatal care.

Our finding is in accordance with previous studies done in several countries as shown in Table 5. The prevalence ranged from 2.4 to 21.53% with RH as the most common ocular findings.

**Table 5:** Retinal hemorrhage prevalence in other countries

Country	Study	Population	Prevalence
China	Li et al. [2]	3573 healthy newborns	21.5% (769/3573)
India	Vinekar et al. [3]	1021 healthy newborns	2.4% (25/1021)
United Kingdom	Callaway et al. [4]	202 healthy newborns	20.3% (41/202)
New Zealand	Simkin et al. [5]	285 healthy newborns	11.8% (33/285)
Indonesia	Present study	1208 healthy newborns	10.9% (132/1208)

Using univariate analysis of risk factors associated with retinal hemorrhage, C-section delivery was shown as a protective factor (OR 0.27, p < 0.001) compared to spontaneous vaginal delivery, while prolonged labor was associated with increased risk of retinal hemorrhage (OR 1.84, p = 0.002). C-section delivery was significantly associated with retinal hemorrhage on multivariate analysis (OR 0.29, p < 0.001) while other risk factors were not. Compared to other studies, C-section delivery has been shown to have a protective factor towards the development of retinal hemorrhage, while spontaneous vaginal delivery increases the risk. Zhao et al. [9] also showed that a history of caesarean delivery is associated with lower rates of retinal hemorrhage (OR 0.296, p value 0.002), meanwhile a spontaneous vaginal delivery is associated with higher risk of developing retinal hemorrhage (OR 4.909, p value < 0.001). A systematic review by Watts et al. [10] demonstrate similar result.

The underlying mechanism of retinal hemorrhage in neonates following spontaneous vaginal delivery was proposed by Yanli et al. [11]. During vaginal delivery, the intracranial pressure rises suddenly due to the compression of the fetal head when the fetus descends. This is accompanied by increased pressure in the central retinal vein, dilatation of the scalp and intracranial veins simultaneously due to venous return obstruction. When this mechanism occurs in newborns whose vascular walls were thinner, they may rupture easily causing RH.

In prolonged labor, especially during the second stage of labor, the uterine contractions are longer, the cervix is fully dilated, and the fetal head had descended. It is possible that compression of the umbilical cord causes intrauterine ischemia and hypoxia. Hypoxic condition may create an acidic environment, which excites the vagus nerve, accelerating bowel movement thus contaminating the amniotic fluid with meconium, which can cause further fetal hypoxia [11]. Hypoxia may cause an autoregulatory cerebral vasodilatation that may lead to increased intracranial pressure, which eventually leads to an increased risk of retinal hemorrhage in the fragile vascular walls of newborns [12].

Considering instrument-assisted delivery, our study showed different results compared with previous studies. In our population, neither vacuum extraction nor forceps deliveries were statistically associated with RH (OR 0.561, p value 0.56). This result was not in accordance to a previous study from Watts et al. [10], which demonstrated that using instruments during delivery increases the incidence of RH (OR 1.75, p = 0.0002). Our result may be due to the low rate of instrument-assisted delivery used in our study population (4.1%). Thus, our findings may not show the exact relationship between instrument-assisted deliveries and the incidence of retinal hemorrhage.

We could not follow up 51 of 132 neonates with RH completely until the end of study. Of these infants, 51% (26 of 51 neonates) had severe RH (grade 3) and the remaining 49% had grade 2 RH. It is generally understood that retinal hemorrhages will resolve quickly during the first few weeks [7], however prolonged macular hemorrhage can create a long-term impact on visual function [13]. It is not yet established if retinal hemorrhage and especially macular hemorrhage and prolonged hemorrhage absorption in neonates would affect eye development or visual function in the long run and further longitudinal studies would be required. However, early diagnosis, monitoring and treatment of retinal hemorrhage would be greatly beneficial to ensure good eye development.

Several important ocular abnormalities may be overlooked as universal eye screening is not yet an established practice. We found other ocular abnormalities, such as intraocular tumor (one case of suspected retinoblastoma) and chorioretinitis (seven cases) which required further immediate workup and appropriate therapy. On average, unilateral retinoblastoma is diagnosed at 25 months while bilateral retinoblastoma is diagnosed at 15 months. Only with known family history would children be screened within the first year after birth [14]. With the implementation of universal eye screening, early diagnosis and treatment of retinoblastoma would be visual-saving and live-saving for patients who would otherwise have poorer prognosis. Early detection and treatment of chorioretinitis is also crucial as serologic testing is not part of standard antenatal care in Indonesia and diagnosis could be missed or delayed resulting in loss of visual function.

In the year 2020, there were 4,771,210 live births [15] with infant mortality rate of 15.9 per 1000 live births [16], which amounts to 4,695,347 surviving infants. Our study found that 12.4% of newborn infants (150 out of 1280) had ocular abnormalities, when extrapolated to a national level, approximates that as many as 580 thousand infants could have ocular abnormalities. Hence, universal eye screening should be a part of mandatory newborn screening in Indonesia to ensure good eye development of infants and early treatment of visual-threating and life-threatening conditions. Our study did not cover some issues regarding the cost-effectiveness of universal eye screening, such as financial, health personnel, and logistics. A separate study would be required to address these issues prior to establishing a universal eye screening as a program at an institutional, or a national level. Other limitations include the lack of data on the antenatal care of the mothers and the unavailability of the follow-up laboratory assessments.

## Conclusion

Our study showed that several important ocular abnormalities can be detected through a universal eye screening in healthy neonates. Retinal hemorrhage is the most common ocular abnormality and is associated with the methods of delivery and the duration of labor. As the ocular abnormalities may go unnoticed during the standard practice, a universal eye screening program should be established especially among those with prolonged labors.

## Data Availability

The datasets used and/or analyzed during the current study are available from the corresponding author on reasonable request.

## Abbreviations

C-section: Caesarian section surgery
CM Hospital: Cipto Mangunkusumo National Referral Hospital
OR: Odds ratio
RH: Retinal hemorrhage
RRT: Red reflex testing
SPSS: Statistical package for the social sciences (computer program
